# Identification of novel HIV-1-derived HLA-E-binding peptides

**DOI:** 10.1016/j.imlet.2018.08.005

**Published:** 2018-10

**Authors:** Zara Hannoun, Zhansong Lin, Simon Brackenridge, Nozomi Kuse, Tomohiro Akahoshi, Nicola Borthwick, Andrew McMichael, Hayato Murakoshi, Masafumi Takiguchi, Tomáš Hanke

**Affiliations:** a The Jenner Institute, Nuffield Department of Medicine, University of Oxford, Oxford, United Kingdom; b Center for AIDS Research, Kumamoto University, Kumamoto, Japan; c NDM Research Building, Nuffield Department of Medicine, University of Oxford, Oxford, United Kingdom; d International Research Center for Medical Sciences, Kumamoto University, Kumamoto, Japan

**Keywords:** HLA-E bindning peptides, HIV-1

## Abstract

Non-classical class Ib MHC-E molecule is becoming an increasingly interesting component of the immune response. It is involved in both the adaptive and innate immune responses to several chronic infections including HIV-1 and, under very specific circumstances, likely mediated a unique vaccine protection of rhesus macaques against pathogenic SIV challenge. Despite being recently in the spotlight for HIV-1 vaccine development, to date there is only one reported human leukocyte antigen (HLA)-E-binding peptide derived from HIV-1. In an effort to help start understanding the possible functions of HLA-E in HIV-1 infection, we determined novel HLA-E binding peptides derived from HIV-1 Gag, Pol and Vif proteins. These peptides were identified in three independent assays, all quantifying cell-surface stabilization of HLA-E*01:01 or HLA-E*01:03 molecules upon peptide binding, which was detected by HLA-E-specific monoclonal antibody and flow cytometry. Thus, following initial screen of over 400 HIV-1-derived 15-mer peptides, 4 novel 9-mer peptides PM9, RL9, RV9 and TP9 derived from 15-mer binders specifically stabilized surface expression of HLA-E*01:03 on the cell surface in two separate assays and 5 other binding candidates EI9, MD9, NR9, QF9 and YG9 gave a binding signal in only one of the two assays, but not both. Overall, we have expanded the current knowledge of HIV-1-derived target peptides stabilizing HLA-E cell-surface expression from 1 to 5, thus broadening inroads for future studies. This is a small, but significant contribution towards studying the fine mechanisms behind HLA-E actions and their possible use in development of a new kind of vaccines.

## Highlights

• 4 novel HIV-derived HLA-E-binding peptides were identified.
• New tools to study HLA-E function.
• Opens a possibility for a new kind of vaccines with superior efficacy, which HIV-1 has not learnt to escape.

## 1 Introduction

Human leukocyte antigen E (HLA-E) is a non-classical major histocompatibility complex (MHC) class Ib molecule, which engages natural killer (NK) and CD8^+^ T lymphocytes and regulates their effector activities. On NK cells, HLA-E molecules contact predominantly C-type lectin-like CD94/NKG2 receptors [1], of which CD94/NKG2A produces inhibitory [2] and CD94/NKG2C activatory [3] signals. CD8^+^ T lymphocytes recognize HLA-E/peptide complexes via their αβ T-cell receptor (TCR) [4], although a small subset also expresses CD94/NKG2A/C [5].

Classical HLA class Ia molecules are highly polymorphic. As of April 2018, there have been 4081 (2853), 4950 (3582) and 3685 (2550) HLA-A, -B and -C alleles (proteins) described, respectively, which help immune cells discriminate between ‘self’ and ‘foreign’ targets. In contrast, only 27 non-classical HLA-E alleles with 8 expressed proteins are known to date, of which 2 have been shown to contribute to immune function [6]. These are HLA-E*01:01 and HLA-E*01:03 and differ in having Arg and Gly in position 107 of the α2-domain outside of the peptide-binding groove, which were associated with low and high expression levels, respectively [7]. Under normal circumstances, HLA-E binds nonameric peptides VMAPRT(L/V)(V/L/I/F)L, here collectively designated VL9, derived from amino acid positions 3–11 of HLA-A, -B, -C and -G leader sequences. Some HLA-B leaders contain Thr in position 2 (p2), which caused aberrant folding, resulted in low or no HLA-E expression [8] and consequently affected engagement with the T-cell and NK receptors [1,2,[9], [10], [11], [12], [13], [14]]. CD8^+^ T cells also recognized HLA-E-presented peptides derived from persistent infections with *Mycobacterium tuberculosis* [15,16], *Salmonella enterica* [17,18] hepatitis C virus [19,20], Epstein-Barr virus [21] and human cytomegalovirus [22], where HLA-E is upregulated to evade NK activity. In the absence of VL9, peptides bound to HLA-E were highly diverse and derived from self-proteins [23,24] with no obvious binding motif and length of up to 18 amino acids [[23], [24], [25]], although the true HLA-association of the very long peptides identified using tandem-mass-spectrometry workflows remains to be verified [26].

Structurally, HLA-E was suggested to be more rigid than the more polymorphic and plastic classical HLA class Ia molecules leaving its groove open in the absence of peptides in contrast to e.g. HLA-A*02:01, the groove of which without a peptide collapsed inwards. The structural analyses of both HLA-E*01:01 [1,12,[27], [28], [29]] and HLA-E*01:03 [30] were limited to the nonameric VL9 peptide variants and suggested major anchor residues at positions p2, p7 and p9. While VL9 bound on the bottom of the HLA-E groove, filled all the binding pockets for hydrophobic amino acid sidechains and formed multiple hydrogen bonds to the peptide backbone [12], ‘atypical’ peptides likely bind in many different ways perhaps higher in the HLA-E groove [31], which may facilitate peptide exchange. Several longer peptides were modelled to budge in the middle part towards the effector-cell receptor [23,24]. These longer peptides required much higher concentrations than VL9 to stabilize HLA-E on the cell surface.

Unexpected MHC-E involvement was found following administration of fibroblast-adapted molecular clone 68-1 of rhesus cytomegalovirus (RhCMV)/simian immunodeficiency virus (SIV)-vectored vaccine, which induced and maintained persistent SIV-specific effector-memory T cells. When challenged, approximately 50% of vaccinated macaques controlled pathogenic SIVmac239 infection and cleared completely the challenge virus [[32], [33], [34]], whereby all macaques became infected and either controlled the challenge virus early or not at all. This protection has now been observed in over 130 animals and required precisely all the mutations present in the RhCMV 68-1 clone. Vaccinated animals had CD8^+^ T cells recognizing rhesus macaque (*Macaca mulatta*) MHC (Mamu) class II- and Mamu-E-restricted epitopes with no classical Mamu class Ia responses and no anti-Env antibodies [35]. These unusual CD8^+^ T-cell responses were present in all protected and unprotected animals. Although to date, there are no formally proven correlates for this vaccine protection, it is hypothesized that the broadly specific Mamu-E presentation contributed to this remarkable outcome. Note that although there is a functional conservation of MHC-E immunobiology among rhesus and cynomolgus macaques and humans [36], 68-1 RhCMV/SIV vaccine only protected rhesus, but not cynomolgus macaques.

The role of HLA-E in regulation of HIV-1 infection remains understudied and the knowledge ‘scattered’. Thus, HIV-1 infection was shown to increase expression of HLA-E and impair NK lysis [37]. HLA-E allelic forms influenced the course of HIV-1 infection, whereby HLA-E*01:03 was associated with 4-fold decreased risk of HIV-1 acquisition in Zimbabwean women [38]. In the same report, women carrying the combination of the protective HLA-E*01:03 homozygote and HLA-G*01:05 heterozygote genotypes had a 12.5-fold decreased risk of HIV-1 infection relative to women carrying neither genotype [38]. HLA-B can be divided into Bw4 and Bw6 based on variation in region 77–83 of the α1-domain of the peptide-binding groove [9], whereby all Bw4 molecules with exception of B*38 carry Thr in p2 of VL9, while Bw6 carries either Thr or Met [39]. Homozygosity of Bw4 (p2 Thr) was associated with lower HIV-1 plasma viral load (pVL) and slower development of AIDS [40], and decreased transmission in heterosexual couples [41]. Separately, HLA-B leader peptide polymorphism was reported to influence the HIV-1 acquisition rate, but not pVL [42]. Thus, further studies of HLA-E role(s) in response to HIV-1 infection are warranted.

To date, there has been only one HIV-1 peptide AISPRTLNA (AA9) reported to be presented by HLA-E [37]. Here, we inform the studies of HLA-E presentation in HIV-1 infection by identification of 4 definite and 5 candidate novel HIV-1-derived minimal peptides capable of binding to HLA-E*01:03 and discuss these results in the context of HLA-E-targeted vaccine and immunotherapeutic modalities.

## 2 Materials and methods

### 2.1 Cryopreserved human PBMCs

The PBMCs were collected in trial HIV−CORE 002 [43,44], which was approved by the National Research Ethics Service Committee West London (Ref: 10/H0707/52) and the UK Medicines and Healthcare products Regulatory Agency (Ref: 21584/0271/001). Volunteers 409 and 410 gave written informed consent before participation. Cryopreserved PBMC samples were stored and used in compliance with the UK Human Tissue Act 2004 and with approval from local NREC.

Cryopreserved PBMCs from 32 treatment-naïve Japanese individuals chronically infected with HIV-1 subtype B were recruited from the National Center for Global Health and Medicine. All volunteers gave written informed consent before participation. The study was approved by the ethics committees of Kumamoto University and the National Center for Global Health and Medicine. Informed consent was obtained from all individuals according to the Declaration of Helsinki. Plasma and PBMCs were separated from whole blood. HLA types of the individuals were determined by standard sequence-based genotyping.

### 2.2 Peptides and antigens

15-mer peptides overlapping by 11 amino acids and spanning the entire first (HIVconsv) and second (tHIVconsvX) generation conserved vaccine immunogens and designated HCXXX and CXXX, respectively, were generously provided by the International AIDS Vaccine Initiative. All 15-mer (GenScriptHK, Hong Kong) and 9-mer (Ana-Spec, San Jose, USA) peptides were at least 90% pure and were reconstituted to 10–40 mg/ml in DMSO, diluted to working stock solutions of 40 mg/ml in PBS and used in mapping studies at final concentration of 100 μg/ml unless otherwise stated.

### 2.3 HLA-expressing cell lines

B-lymphoblastoid cell line **(**LCL) 721.221 cells expressing HLA-E*01:01, E*01:03, A*30:02, B*18:01, B*57:03, C*07:01, C*18:02, A*01:01, A*03:01, B*07:02, B*08:01, C*07:01 and C*07:02 were generated by transfecting the genes into the LCL721.221 cell line and RMA-S cells expressing HLA-E*01:01 and E*01:03 were generated by transfecting the genes into the RMA-S cell line as previously described [45,46]. All cell lines were cultured in RPMI 1640 medium containing 10% FCS medium (R10) with 0.15 mg/ml hygromycin B.

### 2.4 Generation of short-term cell lines (STCL)

Cryopreserved PBMCs were thawed and cultures set up at 1–3 × 10^6^ cells/ml in culture medium containing 1.5 μg/ml 15-mer ‘parental’ peptide and 25 ng/ml IL-7 (R & D Systems). Cultures were supplemented with 100 IU/ml IL-2 (R&D Systems) on day 3, and IL-2 plus fresh culture medium on day 7. On day 10, the cells were washed three times with culture medium, re-suspended in 1 ml of medium and placed at 37 °C, 5% CO_2_ for 48 h to rest prior to the ICS assay.

### 2.5 Intracellular cytokine staining (ICS) assay

LCL 721.221 cell lines expressing a single HLA allele were prepulsed with corresponding peptides, added to the STCLs in a 96-well plate at 37 °C for 2 h. Then, brefeldin A at 10 μg/ml was added for 4 h, the cells were fixed with 4% paraformaldehyde, staining with allophycocyanin (APC)-labeled anti-CD8 mAb (Dako, Glostrup, Denmark), incubated in permeabilization buffer (0.1% saponin, 10% FBS, PBS) and stained with fluorescein isothiocyanate (FITC)-labeled anti-interferon (IFN)-γ mAb (BD Bioscience, CA). Stained cells were acquired using a CyAn ADP Analyser (Beckman Coulter) and analyzed using FlowJo (TreeStar).

### 2.6 HLA-E/peptide stabilization (binding) assay

LCL 721.221 or RMA-S cells transfected with individual HLA-E molecules were grown overnight at 26 °C. Cells were then incubated with the test peptides at 26 °C for 1 h and transferred to 37 °C for 3 h for ‘destaining’. VL9 peptides were incubated at 37 °C for 3 and 24 h. Cell-surface expression of HLA-E*01:01 and HLA-E*01:03 was determined by incubation with PE-conjugated anti-HLA-E antibody, washed twice with PBS and fixed in 100 μl of Cytofix (BD Biosciences). Control cultures were kept at 26 °C for over 16 h, designated ‘for ever’. For assays in Table 3, serum-free medium AIM V (Thermo Fisher Scientific) was used. Cells were acquired using a CyAn ADP Analyser (Beckman Coulter) and analyzed using FlowJo (TreeStar). Results were expressed either directly as mean fluorescent intensity (MFI) or a binding index was calculated as follows: Expression Index = (37 °C with peptide – 37 °C no peptide) / (26 °C with peptide ‘for ever’ – 26 °C no peptide).

### 2.7 Single chain timer-stabilization assay

The chimeric genes coding for the single-chain peptide-β_2_-microblobuline-MHC-E trimers were constructed as described previously [47,48]. Briefly, the DNA fragment coding the tested peptide was inserted downstream of the fragment coding for the signal sequence of HLA-E*01:01 followed by coding sequences for a flexible linker [G_4_S]_4_, β_2_-microglobulin and the desired heavy chain of MHC-E. The Ala at position 84 was mutated to Tyr to accommodate for the flexible linker. The assembled plasmids were then transfected into HEK 293 T cells maintained between 10% and 90% confluency at 37 °C, 5% CO_2_ in DMEM (Life Technologies) supplemented with 10% FBS (SeraLabs), and 50 units/ml Penicillin/50 μg/ml Streptomycin (Life Technologies). Transfections were carried out in 6-well plates using GeneJuice (Millipore) as per the manufacturer’s instructions. One million HEK 293T cells were stained in 100 μl of PBS at 4 °C for 15 min with primary antibody 3D12 for HLA-E (BioLegend) or 4D12 for Mamu-E (MBL), washed twice with PBS, stained with secondary antibody APC-conjugated goat-anti-mouse (H + L) F(ab')_2_ fragment (Life Technologies), washed as before and fixed in 100 μl of Cytofix (BD Biosciences). Stained cells were acquired using a CyAn ADP Analyser (Beckman Coulter) and analyzed using FlowJo (TreeStar).

## 3 Results

### 3.1 PM9 is an optimal peptide for HLA-B*18:01 and an HLA-E*01:03 binder

The first-generation candidate T-cell vaccine against HIV-1 employing conserved regions of the HIV-1 proteome [49] was tested in HIV-1-negative adults in trial HIV−CORE 002 and induced robust HIV-1-specific T-cell responses. These responses were detected by pools of 15-mer peptides overlapping by 11 amino acids across the full length of the HIV-1-derived vaccine immunogen [50]. Following a routine mapping of stimulatory 15-mer peptides and narrowing to optimal epitopes recognized by vaccine-elicited CD8^+^ T lymphocytes, peptide PEIVIYDYM (PM9) was identified, which was targeted by vaccine recipient 410 (A*30:02, B*18:01, B*57:03, C*07:01, C*18:02) and presented by HLA-B*18:01 [51]. In particular, HLA restriction was determined by expanding volunteer’s PBMC with stimulatory ‘parental’ peptide FRAQNPEIVIYQYMD**KK** (HC092) (Lys’s shown in bold were added for peptide solubility) to establish a 10-day short-term cell line (HC092 SCTL). HC092 SCTL was then assayed in an intracellular cytokine-staining (ICS) assay against PM9-pulsed LCL721.221 cells untransfected or stably expressing the six volunteer’s HLA class I molecules. Note that LCL721.221 cells are of human origin, do not express the HLA–A and -B molecules, but express HLA-C*01:02 and carry genes coding for HLA-E*01:01 and HLA-G [52]. We confirmed that HLA expression by stably transfected LCL721.221 cells alone, i.e. in the absence of the PM9 peptide, increased the surface expression of HLA-E*01:01 (Table 1). While the strongest IFN-γ production of 28.3% by CD8^+^ HC092 STCL cells was clearly induced with the HLA-B*18:01 transfectant, all other tested cultures including untransfected LCL721.221 cells stimulated between 15.1% and 18.1% of CD8^+^ STCL and did so in a PM9-dependent manner (Fig. S1). Similar baseline stimulation was not observed with other tested peptides. While we were never able to unequivocally establish whether or not the TCR-HLA-E*01:01/PM9 interaction was responsible for stimulation of these CD8^+^ STCL cells, it prompted us to test the PM9 peptide for binding to HLA-E.

**Table 1:** Stable transfection of LCL721.221 cells with classical HLA class I molecules increased cell-surface expression of HLA-E*01:03.

		37 °C MFI^a^	
Transgene	VL9	3 hours	24 hours
HLA-A*02:01	VMAPRTLVL	12.7	2.90
HLA-B*08:01	VMAPRTVLL	16.3	2.66
HLA-B*51:01	VTAPRTVLL	3.92	2.69
HLA-C*03:03	VMAPRTLIL	8.38	2.62
HLA-C*07:01	VMAPRALLL	7.21	2.52
CD4	–	4.16	2.39

a LCL721.221 cells were stably transfected with various HLA alleles or CD4 and incubated overnight at 26 °C. The cultures were then transferred to 37 °C for either 3 or 24 h and their cell surface expression of HLA-E*01:01 was assessed by mAb 3D12 using FACS analysis. The gating strategy was similar to Fig. S2, except that transfected LCL721.221 cells were used.

**Fig. 1:**
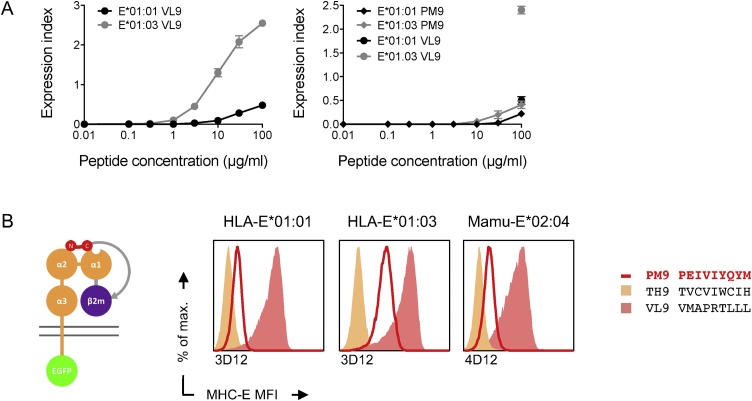
PEIVIYQYM (PM9) is a new HIV-1–derived peptide binding HLA-E. (A) RMA-S cells stably expressing HLA-E*01:01, HLA-E*01:03 or HLA-B*18:01 were pulsed with increasing concentration of either PM9 or VL9 peptides and the cell-surface stabilization of the HLA-E and HLA-B molecules were determined by using mAbs 3D12 and W6/32, respectively. Please see gating strategy in Fig. S2. (B) A single chain peptide-β_2_-microglobuline-MHC-E heavy chain trimer (left) cell-surface stabilization was used to test candidate peptides for binding to MHC-E. Briefly, DNA fragments coding for the tested peptides were inserted into a plasmid for expression of the entire trimer in one open-reading frame. This plasmid DNA was transiently transfected into HEK 293T cells and the trimer surface expression (red line) was detected by antibodies indicated below the graphs using flow cytometry. Stabilization by VL9 (yellow area) and irrelevant (pink area) peptide were used as positive and negative controls. Please see gating strategy in Fig. S3.

Binding of PM9 to both HLA-E*01:01 and –E*01:03 alleles was assessed using an HLA-E/peptide cell-surface stabilization assays. Thus, murine RMA-S cells deficient in transporter associated with antigen processing (TAP) were stably transfected with one of the two HLA-E alleles together with human β_2_-microglobuline. Then, stabilization of HLA-E/PM9 and HLA-E/VL9 complexes was compared in peptide-titration analyses using peptide concentrations ranging from 0.01 μg/ml to 100 μg/ml by growing transfected RMA-S cells overnight at 26 °C, pulsing them with peptide for 1 h and transferring them to 37 °C for 3 additional hours. Weak half-maximal effective concentrations (EC_50_) of over 100 μM were indicated for the interaction of PM9 with the two HLA-E*01:01 and E*01:03 molecules (Fig. 1A).

To further confirm PM9-interaction with the MHC-E molecules, we employed an assay based on cell-surface stabilization of single-chain peptide-β_2_-microblobuline-MHC-E trimers [47]. Briefly, a DNA fragment coding for the tested peptide, here PM9, was inserted downstream of an HLA-E leader peptide and was followed by flexible [G_4_S]_4_ linker, β_2_-microblobuline and Y84 A point-mutated MHC-E heavy chain, which accommodated the flexible linker (Fig. 1B left). The corresponding plasmid was transiently transfected into HEK 293T cells and a successful peptide-MHC-E interaction resulted in an increased cell-surface expression of assembled single-chain trimmers. Thus, using HLA-E*01:01, HLA-E*01:03 and Mamu-E*02:04, expression of the PM9 peptide increased the mean fluorescent intensity (MFI) relative to a non-binding peptide TVCVIWCIH (TH9) for all three employed MHC-E molecules. The highest increase was observed for E*01:03 and reached approximately half of the VL9 MFI (Fig. 1B). From these experiments, we concluded that the PM9 peptide bound to HLA-E molecules, and stabilized and increased the HLA-E expression on the cell surface.

### 3.2 HC102/HC103 overlapping peptides share HLA-A*03:01 epitope and HLA-E*01:03 binder RV9

**Fig. 2:**
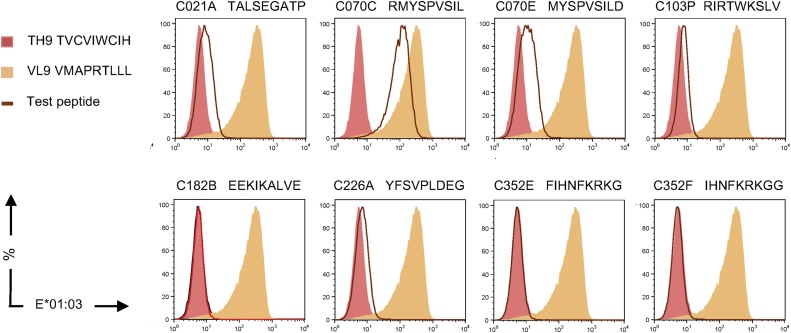
Identification of HLA-E-binding peptides in a single-chain trimer stabilization assay. A single chain peptide-β_2_-microglobuline-MHC-E heavy chain trimer stabilization assay was employed to determine the candidate 9-mer peptide binding to HLA-E*01:03 (brown line) and compared to that induced by VL9 (yellow area) and irrelevant (pink area) peptide. Expression of trimer on transiently transfected into HEK 293T cells was assessed using a chromogen-conjugated 3D12 mAb in flow cytometry. Please see gating strategy in Fig. S3.

STCL were expanded from PBMC of vaccine recipient 409 (A*01:01, A*03:01, B*07:02, B*08:01, C*07:01, C*07:02) using peptides **K**QVDRMRIRTWKSLVK (HC102) and MRIRTWKSLVKHHLT (HC103). These HC102 and HC103 STCLs were then stimulated with LCL721.221 expressing individually all six volunteer’s HLA class I molecules and pulsed with the two ‘parental’ long peptides. The experiments indicated that peptide-pulsed LCL721.221/HLA-A*03:01 stimulated 5.31% and 13.7% of the corresponding CD8^+^ HC102 and HC103 STCLs, respectively, yielded approximately 2-fold higher frequencies than those induced by peptide-pulsed untransfected and other HLA-expressing LCL721.221 cells, and the HLA-E-cell-surface stabilization was peptide dependent (Fig. S4). Again, we were never able to prove that this half-stimulation of CD8^+^ cells was triggered by the TCR-HLA-E*01:01/peptide interaction, but we did establish that peptide RIRTWKSLV (RV9) shared between the HC102 and HC103 parental peptides was capable of stabilizing HLA-E*01:03 on LCL721.221 cells stably overexpressing the HLA-E*01:03 molecule and did so weakly, but more avidly than majority of other tested HIV-1 peptides (see below Table 3.). Weak binding of RV9 to HLA-E*01:03 was also marginally indicated in the single-chain peptide-β_2_-microblobuline-MHC-E trimer assay (Fig. 2).

### 3.3 Identification of HIV-1-derived HLA-E-stabilizing 15-mer peptides

Next, we decided to screen systematically 401 overlapping 15-mer peptides derived from highly conserved regions of HIV-1 proteins employed in the second-generation, conserved-region HIV-1 vaccines [53] for binding to the HLA-E*01:01 molecule on the human LCL721.221 cells and 17 candidates were identified. These 17 15-mer peptides were retested for specific binding to HLA-E*01:01 and -E*01:03-transfected murine RMA-S cells, whereby most of the HLA-E*01:03-stabilizing peptides also increased HLA-E*01:01 expression, but their binding index was approximately 2-fold lower (Table 2).

**Table 2:** Peptides derived from the second-generation conserved-region candidate HIV-1 vaccine binding HLA-E.

		Cell-Surface Stabilization Index^b^			
Name	Sequence^a^	E*01:01	E*01:03 ± SD	E*01:03/VL9^c^	E*01:03/PM9^c^
	**T**ALSEGATPQDLNTM	0.25	0.373 ± 0.055	2.600	0.617
C022	**S**ALSEGATPQDLNMM	0.14	0.382 ± 0.027	2.600	0.617
C025	PQDLN**T**MLNTVGGHQ	0.32	0.431 ± 0.038	2.281	0.620
C026	PQDLN**M**MLNIVGGHQ	0.25	0.429 ± 0.037	2.281	0.620
	GHQAAMQMLK**D**TINE	0.27	0.455 ± 0.033	2.281	0.620
C032	GHQAAMQMLK**E**TINE	0.16	0.480 ± 0.057	2.281	0.620
C037	INEEAAEWDR**V**HPVH	0.30	0.419 ± 0.031	2.281	0.620
C038	INEEAAEWDR**L**HPVH	0.20	0.389 ± 0.039	2.281	0.620
	IVRMYSPVSILDI**R**Q	0.21	0.476 ± 0.088	2.572	0.658
	IVRMYSPTSILDI**K**Q	0.03	0.518 ± 0.033	2.572	0.658
C182	TEEKIKALTEICKEM	0.33	0.463 ± 0.085	2.572	0.658
C226	YFSVPLDE**S**FRKYTA	−0.15	0.669 ± 0.010	2.369	0.733
C227	YFSVPLDK**D**FRKYTA	0.21	0.618 ± 0.042	2.369	0.733
C352	QMAV**F**IHNFKR**K**GGI	0.26	0.641 ± 0.051	2.369	0.733
C353	QMAV**L**IHNFKR**R**GGI	0.17	0.632 ± 0.065	2.369	0.733
C376	VYYRDSRDP**I**WKGPA	0.22	0.366 ± 0.044	2.572	0.658
C377	VYYRDSRDP**L**WKGPA	0.17	0.353 ± 0.039	2.572	0.658

a The immunogens of second generation of the conserved-region candidate T-cell HIV-1 vaccine were designed as a bivalent mosaic [63], which maximizes the perfect match of 9-mer potential T-cell epitopes on the vaccine with the globally circulating HIV-1 variants [53]. Thus, for each selected conserved region of the HIV-1 proteome, which was employed in the vaccine immunogens, two versions of the amino acid sequences were computed, which differed in approximately 1 in 10 amino acids (bold underlined). Exceptionally, the two peptides were identical in both bi-valent mosaic immunogens (e.g. peptide C182).

b The cell-surface stabilization index on RMA-S cells transfected with either HLA-E*01:01 or HLA-E*01:03 together with human β_2_-microglobulin is calculated as follows: Index = (Sample 37 °C – No peptide 37 °C) / (Sample 26 °C – No peptide 26 °C). The results for HLA-E*01:03 are shown as mean $\pm \;SD\;\left(n=3\right).$ See Fig. S2 for the gating strategy. Table shows peptides with stronger binding that the majority of the 401 screened peptides tested on LCL721.221 (HLA-E*01:01), the cell-surface stabilization indices of which were between 0 and 0.01.

c On each experimental day, VL9 (VMAPRTLVL) and PM9 peptides were included as positive controls.

### 3.4 Identification of HIV-1-derived HLA-E*01:03-stabilizing 9-mer peptides

We reasoned that HLA-E likely binds 9-mer peptides with higher affinities relative to the parental 15-mers perhaps through a better fit into the peptide-binding groove of the HLA-E molecules and, therefore, using 9-mers in the binding assay may result in more pronounced surface stabilization of the HLA-E*01:03 molecules. For these binding studies, we employed LCL721.221 cells strongly overexpressing HLA-E*01:03. The 9-mer peptides were designed based on the 15-mer results above. As positive and negative controls, we used the VL9 and no added peptide, which yielded mean ± SD HLA-E MFIs of 14265 ± 512 and 3380 ± 10, respectively. The MFI for the 32 tested HIV-1-derived 9-mers ranged from 2481 ± 93 to 10132 ± 241 with 7 peptides yielding MFI above 4000 (Table 3). Eight 9-mers were also incorporated into the single-chain-trimer, of which 5 peptides RMYSPVSIL (RL9), TALSEGATP (TP9), RIRTWKSLV (RV9), YFSVPLDEG (YG9) and MYSPVSILD (MD9) showed increased HLA-E*01:03 surface stabilization (Fig. 2). In the LCL721.221/HLA-E*01:03 assay, the mean ± SD MFIs of these 5 trimer-stabilizing peptides was 10132 ± 249, 6702 ± 537, 4514 ± 467, 3966 ± 10 and 3313 ± 269, respectively (Table 3).

**Table 3:** Identification of HLA-E binding 9-mer peptides.

LCL 721.221 cells overexpressing HLA-E*01:03 were pulsed with indicated 9-mer peptides and the surface HLA-E*01:03 stabilization was determined using flow cytometry. Expression is shown as MFI. Please see gating strategy in Fig. S5. MFI data are shown as mean ± SD (n = 3). Colour coding: MFIs:  3380–3999;  4000–4999;  5000–5999;  6000–6999;  over 10,000;  over 14,000.  Positive and  no stabilization in the SCT assay. Bold: confirmed HLA-E*01:03 binder peptides in two assays (MFI > 4000). (For interpretation of the references to colour in this table legend, the reader is referred to the web version of this article).

All surface stabilization data are summarized in Table 3. Thus, altogether in this work, we identified 4 novel previously undescribed peptides TP9, RL9, PM9 and RV9 derived from HIV-1 capable of stabilization of HLA-E molecules on the surface of cells in both the SCT assay and over 4000 MFI in the LCL721.221-HLA-E*01:03 assay, and further 5 possible candidate binder peptides NR9, QF9, YG9, EI9 and MD9, which provided a positive stabilization signal in at least one of the two employed assays.

## 4 Discussion

HLA-E is a relatively understudied molecule of the immune system. It can potently inhibit NK killing by presentation of peptides derived from leader sequences of other HLA class I molecules, but much less is known about its other more subtle roles involving CD8^+^ T cells and indeed its peptide repertoire. Immunity is a highly efficient defence system evolved to protect higher organisms against invading microorganisms and cancer, and as such it must be very finely tuned between uncompromising protective responses and self-harm; HLA-E is likely a significant candidate component in maintaining this fine balance as suggested for example by *M. tuberculosis* challenge experiments in genetically modified mice [54]. In the HIV-1 vaccine field, the Mamu-E presentation got into the spotlight by its as-yet-unproven involvement in conferring protection for half of the rhesus macaques challenged with pathogenic SIVmac239 and subsequent complete clearance of SIVmac239 from the body [[32], [33], [34]]. By inference, HLA-E likely plays an important role in balancing the immune responses to HIV-1 infection. However, to date, there has been only one HIV-1-derived epitope AISPRTLNA (AA9) reported to bind HLA-E and inhibit NK cell attack [37]. Here, to start building a toolbox for deciphering the likely multiple intertwined roles of HLA-E in anti-HIV-1 response, we have identified 4 novel HLA-E-stabilizing epitope peptides RMYSPVSIL (RL9), PEIVIYDYM (PM9), TALSEGATP (TP9) and RIRTWKSLV (RV9), and 5 other candidate HLA-E-binding peptides EKIKALVEI (EI9), MYSPVSILD (MD9), NEEAAEWDR (NR9), QMAVFIHNF (QF9) and YFSVPLDEG (YG9) positive in only one of the two assays used with the potential to provide specific signals to effector cells of the immune response.

Two identified HLA-E-binding peptides were optimal epitope peptides for other canonical HLA class I molecules. Thus, PEIVIYDYM (PM9) is an optimal epitope for HLA-B*18:01 [51] and RIRTWKSLV (RV9) is restricted by HLA-A*03:01. A quick database search of HIV-1-derived CD8^+^ T-cell epitopes revealed that the most avidly HLA-E-binding peptide of the new HIV-1 derivatives RMYSPVSIL (RL9) is a human A2 epitope [[55], [56], [57]], predicted to bind TAP and HLA-A*02/A*24/A*31/B*39/B*52 [58,59]. We previously reported activation of CD8^+^ T cells by the IHNFKRKGG (IG9) peptide in two vaccine recipients, but were unable to identify the restricting HLA class Ia element [51]; there is a possibility that, in this case, CD8^+^ T cells were activated through CD94/NKG2C stimulatory receptor present on about 10% of circulating CD8^+^ T lymphocytes [5]. As for the other identified HLA-E binders, none matched precisely as 9-mer any already known optimal epitope, although several near matches of known epitopes were found. Although similarity in peptide-binding motifs between HLA-E and A*02:01 was once suggested [25], the significance of these observations is presently unclear. For LCL721.221 cells, there is a possibility that INF-γ-production by HC092 SCTL and HC102/HC103 STCL could have been induced through the HLA-C*01:02 molecule signaling also expressed on these cells. However, this was not the case for the murine RMS-A cells, hence supporting the HLA-E involvement.

The strongest HLA-E-binding peptides VL9 are strictly 9-mers and the relatively strongly binding non-VL9 peptides are also 9-mers. With the caveat of the possibility of co-purification rather than HLA association [26], up to 17-amino acid-long self-peptides were eluted from soluble HLA-E*01:01 and HLA*E01:03 molecules and sequenced by nanoflow liquid chromatography tandem mass spectrometry (LC–MS/MS) [23,24]. In the past, we similarly eluted longer peptides from classical HLA class Ia molecules [[60], [61], [62]]. In the same study above, Celik et al. found that HLA-E*01:01 and HLA-E*01:03 alleles did not share the same peptidome, nevertheless presented peptides, which were derived from pools of closely related protein subtypes such as histones and ribosomal proteins [23,24]. The MHC-E peptide-binding groove is more open relative to that on canonical class Ia molecules [47] and thus can readily accommodate longer peptides. This also concurs with our identification of 17 HLA-E-stabilizing 15-mer peptides during the initial screen for HIV-1-derived binders (Table 2). A diversity of SIV-derived 15-mer and optimal 9-mer peptides was presented by Mamu-E*02:03, -E*02:11 and –E*02:20 in RhCMV68-1/SIV-vaccinated macaques, whereby on average 4 Mamu-E-binding peptides were identified per every 100 amino acids of SIV protein length as well as ‘supertopes’ recognized by all (n = 130) vaccinated animals [47]. In the present study, the tHIVconsvX immunogens were 873 amino acids long and 9 binding 15-peptide pairs were identified (Table 2). This was approximately a 4-fold lower frequency of HLA-E binders in humans compared to RhCMV68-1/SIV vaccinated macaques, which may reflect the generally broader epitope presentation by rhesus macaques relative to humans. In the same macaque study above, search for amino acids enriched in certain 9-mer positions of 11 optimal mapped epitopes and 551 Mamu-E-eluted, LC–MS/MS sequenced peptides failed to provide any obvious binding motif. As the authors commented, this was unexpected given the limited polymorphism of the Mamu-E molecule [47].

So far, we were unable to confirm existence of vaccine- or natural HIV-1 infection-elicited CD8^+^ T lymphocytes recognizing HIV-1-derived peptides through their TCR and this is not for lack of trying. Our search for PM9-specific HLA-E-restricted CD8^+^ T-cell responses in natural HIV-1 infection identified one possible responder out of 32 patients with a barely detectable response (Fig. S6). Since humans infected with *M. tuberculosis* have readily detectable CD8^+^ T cells specific for HLA-E-presented *M. tuberculosis* peptides, perhaps search for such HLA-E-restricted HIV-1-specific CD8 T cells in dually HIV-1- *and M. tuberculosis* co-infected individuals might yield some interesting results.

HIV-1 vaccine is the best solution to ending the AIDS epidemic. Induction of HLA-E-restricted protective CD8^+^ T-cell responses by vaccination has several attractions such as limited HLA-E polymorphism, which would help deal with HIV-1 global variability and immune evasion. Given the non-human primate protection and a complete clearance of an immunodeficiency virus from the body, HLA-E-specific responses may have superior efficacy to regular HLA class Ia-restricted T cells. Because these responses are not dominantly utilized during natural infection, HIV-1 has not adapted to them. In the present work, we have expanded the knowledge of HIV-1-derived target peptides stabilizing HLA-E cell surface expression.

## Competing interests

Authors declare no competing interests.
