# Supplementary material for: Identification of novel HIV-1-derived HLA-E-binding peptides
Source: Immunol Lett. 2018 Oct;202:65–72. doi: 10.1016/j.imlet.2018.08.005 (PMC6291738; doi:10.1016/j.imlet.2018.08.005)
Supplement: Supplementary file 1 [file mmc1.pptx]

## Slide 1
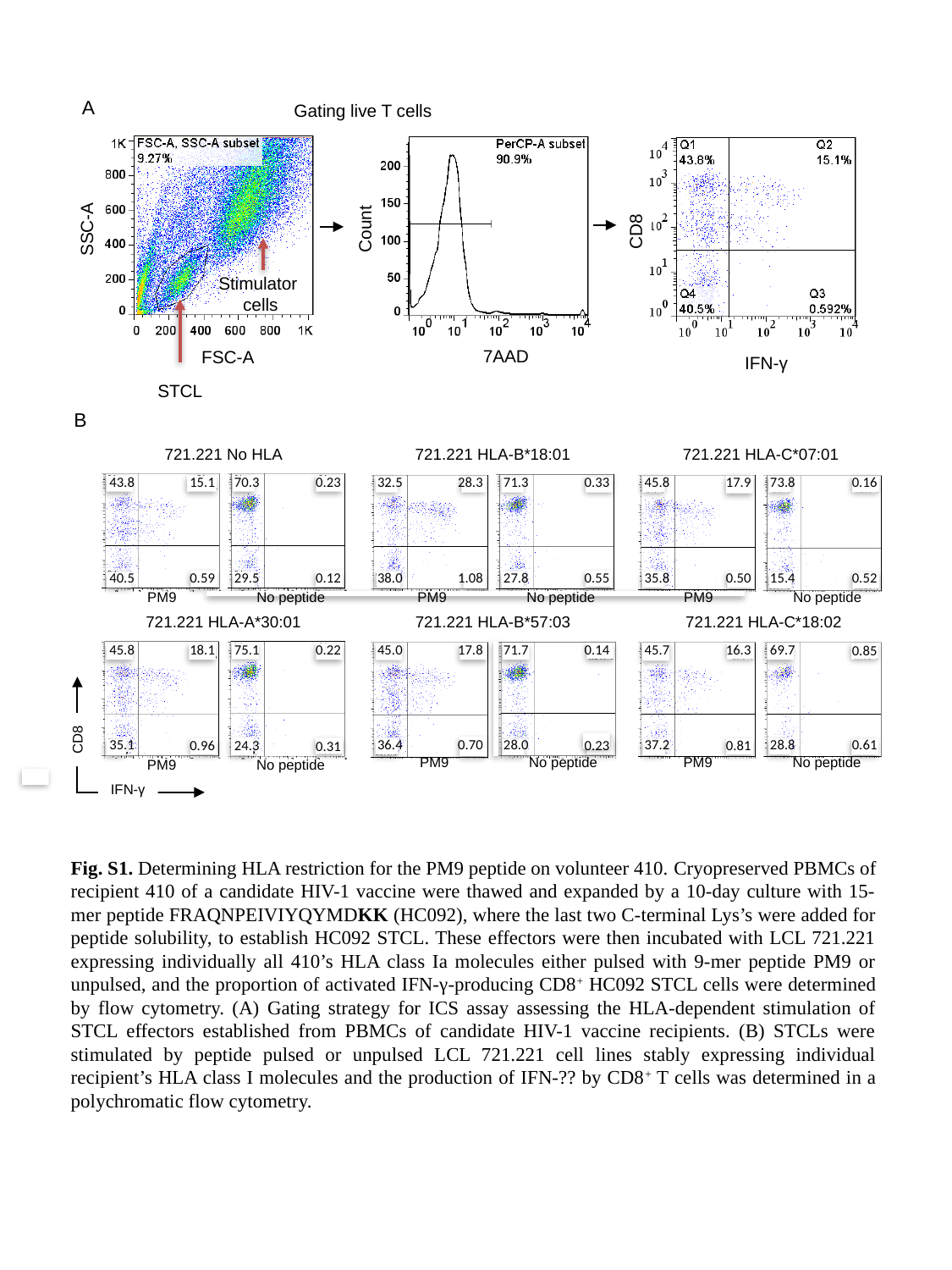

A
Gating live T cells
7AAD
SSC-A
Count
CD8
Stimulator
 cells
FSC-A
IFN-γ
STCL
B
721.221 No HLA
721.221 HLA-B*18:01
721.221 HLA-C*07:01
43.8
15.1
70.3
0.23
32.5
28.3
71.3
0.33
45.8
17.9
73.8
0.16
40.5
0.59
29.5
0.12
38.0
1.08
27.8
0.55
35.8
0.50
15.4
0.52
PM9 No peptide
PM9 No peptide
PM9 No peptide
721.221 HLA-A*30:01
721.221 HLA-B*57:03
721.221 HLA-C*18:02
75.1
0.14
45.8
18.1
0.22
45.0
17.8
71.7
45.7
16.3
69.7
0.85
CD8
IFN-γ
35.1
36.4
0.70
28.0
37.2
28.8
0.61
0.96
24.3
0.23
0.81
0.31
PM9 No peptide
PM9 No peptide
PM9 No peptide
Fig. S1. Determining HLA restriction for the PM9 peptide on volunteer 410. Cryopreserved PBMCs of recipient 410 of a candidate HIV-1 vaccine were thawed and expanded by a 10-day culture with 15-mer peptide FRAQNPEIVIYQYMDKK (HC092), where the last two C-terminal Lys’s were added for peptide solubility, to establish HC092 STCL. These effectors were then incubated with LCL 721.221 expressing individually all 410’s HLA class Ia molecules either pulsed with 9-mer peptide PM9 or unpulsed, and the proportion of activated IFN-γ-producing CD8+ HC092 STCL cells were determined by flow cytometry. (A) Gating strategy for ICS assay assessing the HLA-dependent stimulation of STCL effectors established from PBMCs of candidate HIV-1 vaccine recipients. (B) STCLs were stimulated by peptide pulsed or unpulsed LCL 721.221 cell lines stably expressing individual recipient’s HLA class I molecules and the production of IFN-?? by CD8+ T cells was determined in a polychromatic flow cytometry.

## Slide 2
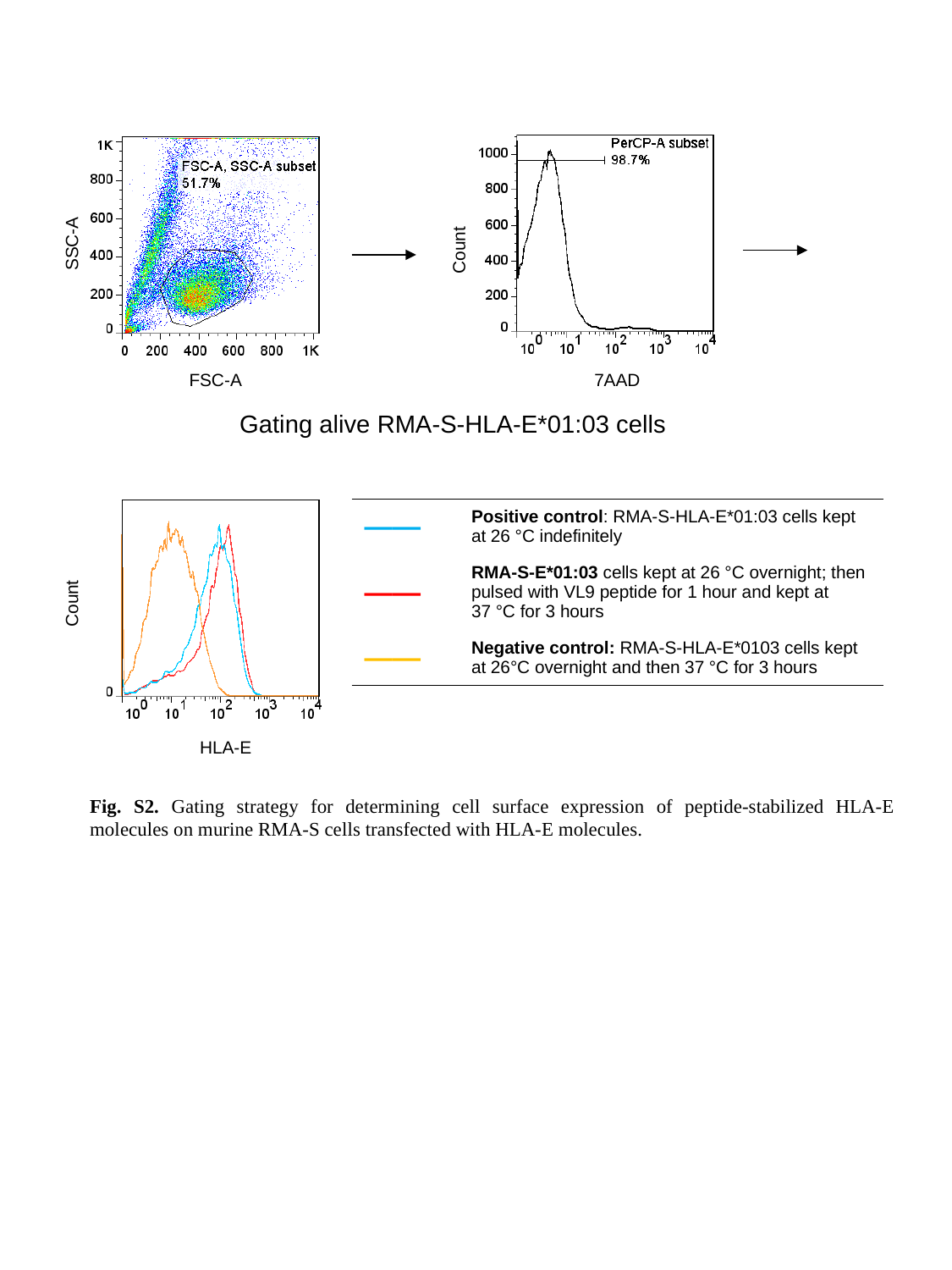

SSC-A
Count
FSC-A
7AAD
Gating alive RMA-S-HLA-E*01:03 cells
| —— | Positive control: RMA-S-HLA-E\*01:03 cells kept at 26 °C indefinitely |
| --- | --- |
| —— | RMA-S-E\*01:03 cells kept at 26 °C overnight; then pulsed with VL9 peptide for 1 hour and kept at 37 °C for 3 hours |
| —— | Negative control: RMA-S-HLA-E\*0103 cells kept at 26°C overnight and then 37 °C for 3 hours |
Count
HLA-E
Fig. S2. Gating strategy for determining cell surface expression of peptide-stabilized HLA-E molecules on murine RMA-S cells transfected with HLA-E molecules.

## Slide 3
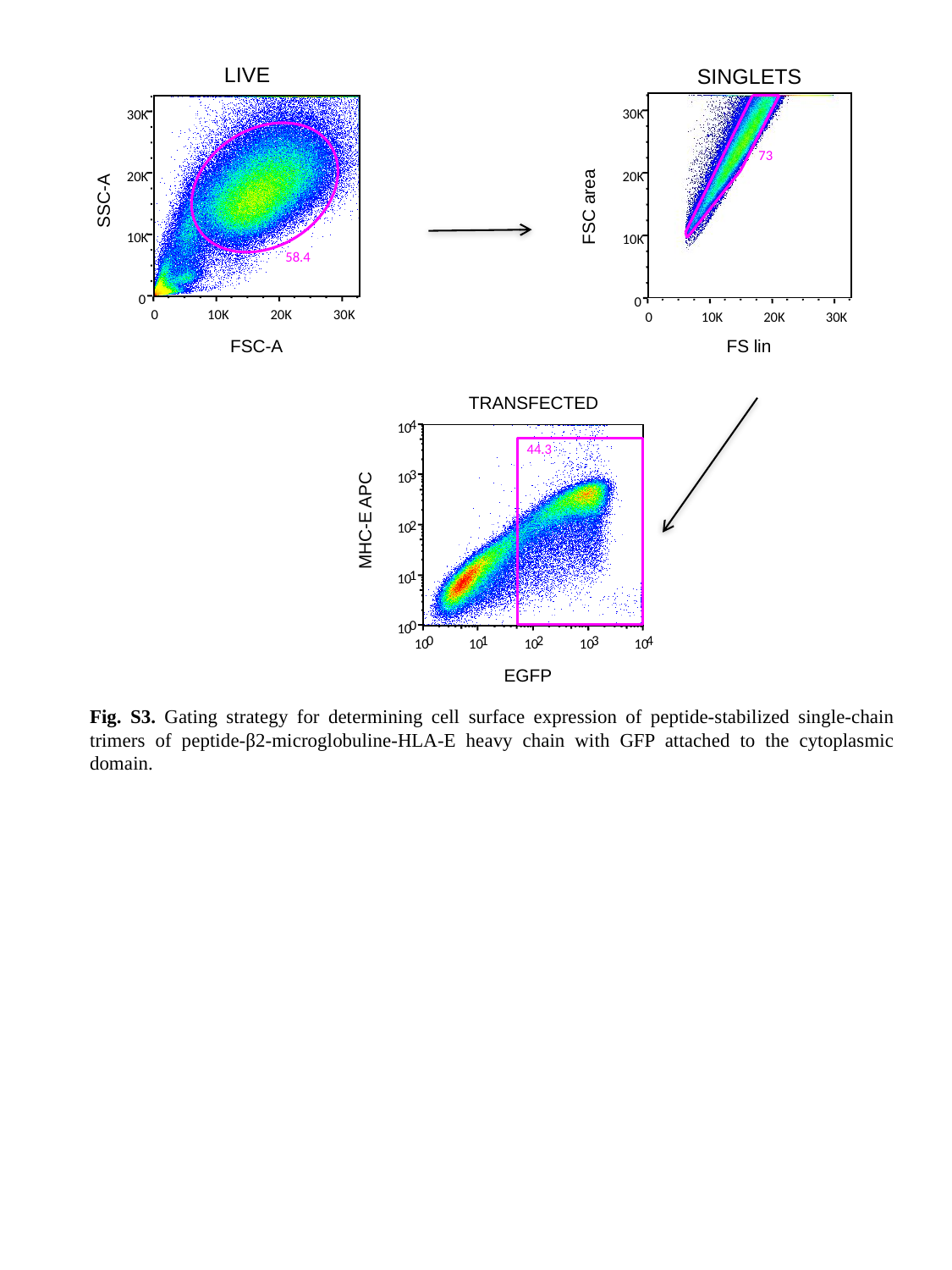

LIVE
30K
20K
10K
0
58.4
0
10K
20K
30K
SINGLETS
30K
20K
10K
0
73
SSC-A
FSC area
0
10K
20K
30K
FSC-A
FS lin
TRANSFECTED
4
10
3
10
2
10
1
10
0
10
0
1
2
3
4
10
10
10
10
10
44.3
MHC-E APC
EGFP
Fig. S3. Gating strategy for determining cell surface expression of peptide-stabilized single-chain trimers of peptide-β2-microglobuline-HLA-E heavy chain with GFP attached to the cytoplasmic domain.

## Slide 4
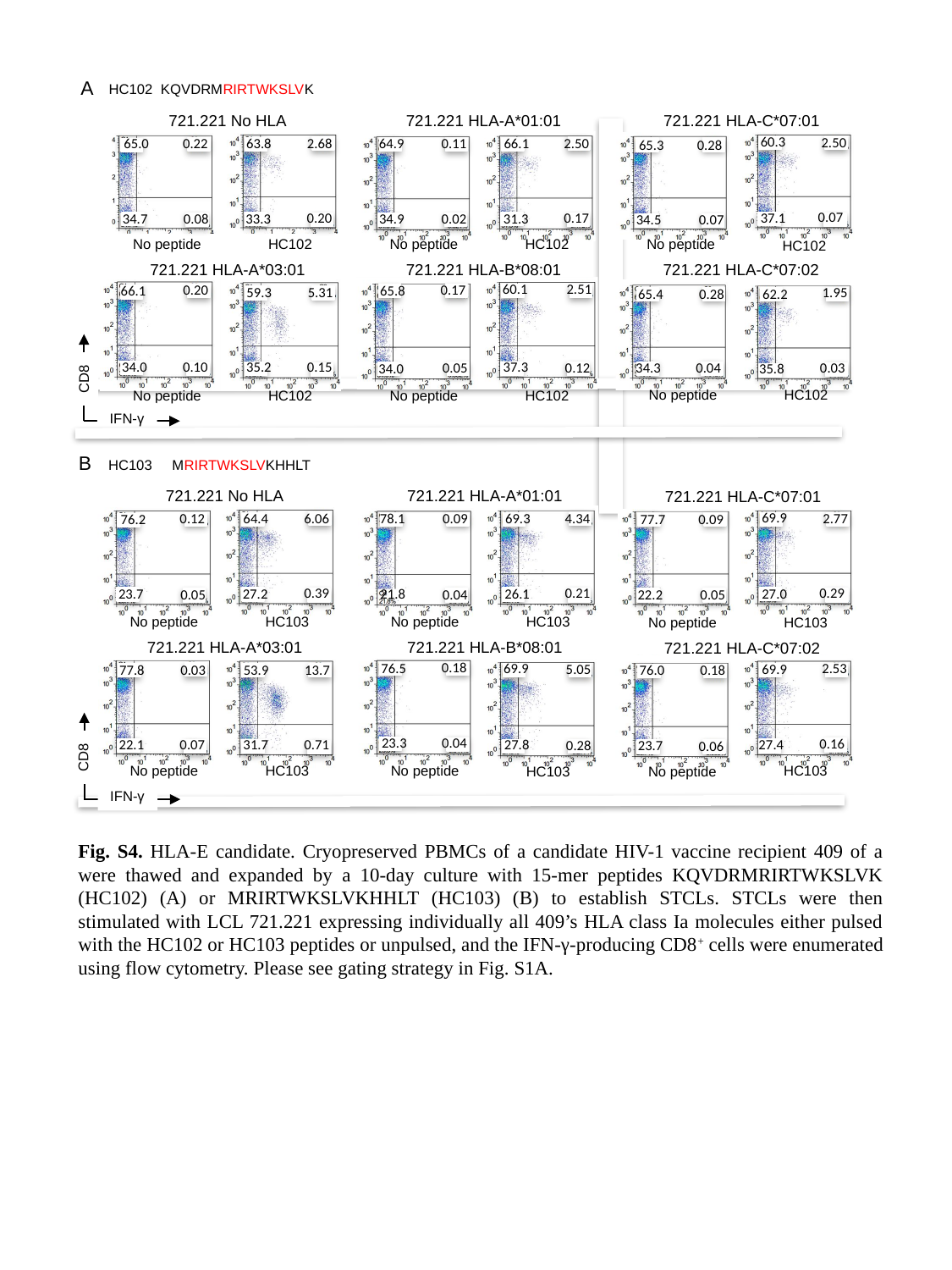

A
HC102 KQVDRMRIRTWKSLVK
721.221 HLA-C*07:01
60.3
2.50
65.3
0.28
0.07
37.1
34.5
0.07
No peptide
HC102
721.221 HLA-C*07:02
1.95
65.4
0.28
62.2
34.3
0.03
0.04
35.8
No peptide
HC102
721.221 No HLA
65.0
0.22
63.8
2.68
0.20
33.3
34.7
0.08
No peptide
HC102
721.221 HLA-A*03:01
0.20
66.1
59.3
5.31
34.0
0.10
35.2
0.15
No peptide
HC102
721.221 HLA-A*01:01
64.9
0.11
66.1
2.50
0.17
34.9
31.3
0.02
No peptide
HC102
721.221 HLA-B*08:01
60.1
2.51
0.17
65.8
37.3
0.05
0.12
34.0
No peptide
HC102
CD8
IFN-γ
B
HC103 MRIRTWKSLVKHHLT
721.221 No HLA
0.12
64.4
6.06
76.2
0.39
23.7
27.2
0.05
No peptide
HC103
721.221 HLA-A*03:01
77.8
0.03
53.9
13.7
22.1
0.07
31.7
0.71
No peptide
HC103
721.221 HLA-A*01:01
78.1
0.09
69.3
4.34
0.21
21.8
26.1
0.04
No peptide
HC103
721.221 HLA-B*08:01
0.18
76.5
69.9
5.05
23.3
0.04
27.8
0.28
No peptide
HC103
721.221 HLA-C*07:01
69.9
2.77
77.7
0.09
0.29
27.0
22.2
0.05
No peptide
HC103
721.221 HLA-C*07:02
2.53
69.9
76.0
0.18
0.16
27.4
23.7
0.06
HC103
No peptide
CD8
IFN-γ
Fig. S4. HLA-E candidate. Cryopreserved PBMCs of a candidate HIV-1 vaccine recipient 409 of a were thawed and expanded by a 10-day culture with 15-mer peptides KQVDRMRIRTWKSLVK (HC102) (A) or MRIRTWKSLVKHHLT (HC103) (B) to establish STCLs. STCLs were then stimulated with LCL 721.221 expressing individually all 409’s HLA class Ia molecules either pulsed with the HC102 or HC103 peptides or unpulsed, and the IFN-γ-producing CD8+ cells were enumerated using flow cytometry. Please see gating strategy in Fig. S1A.

## Slide 5
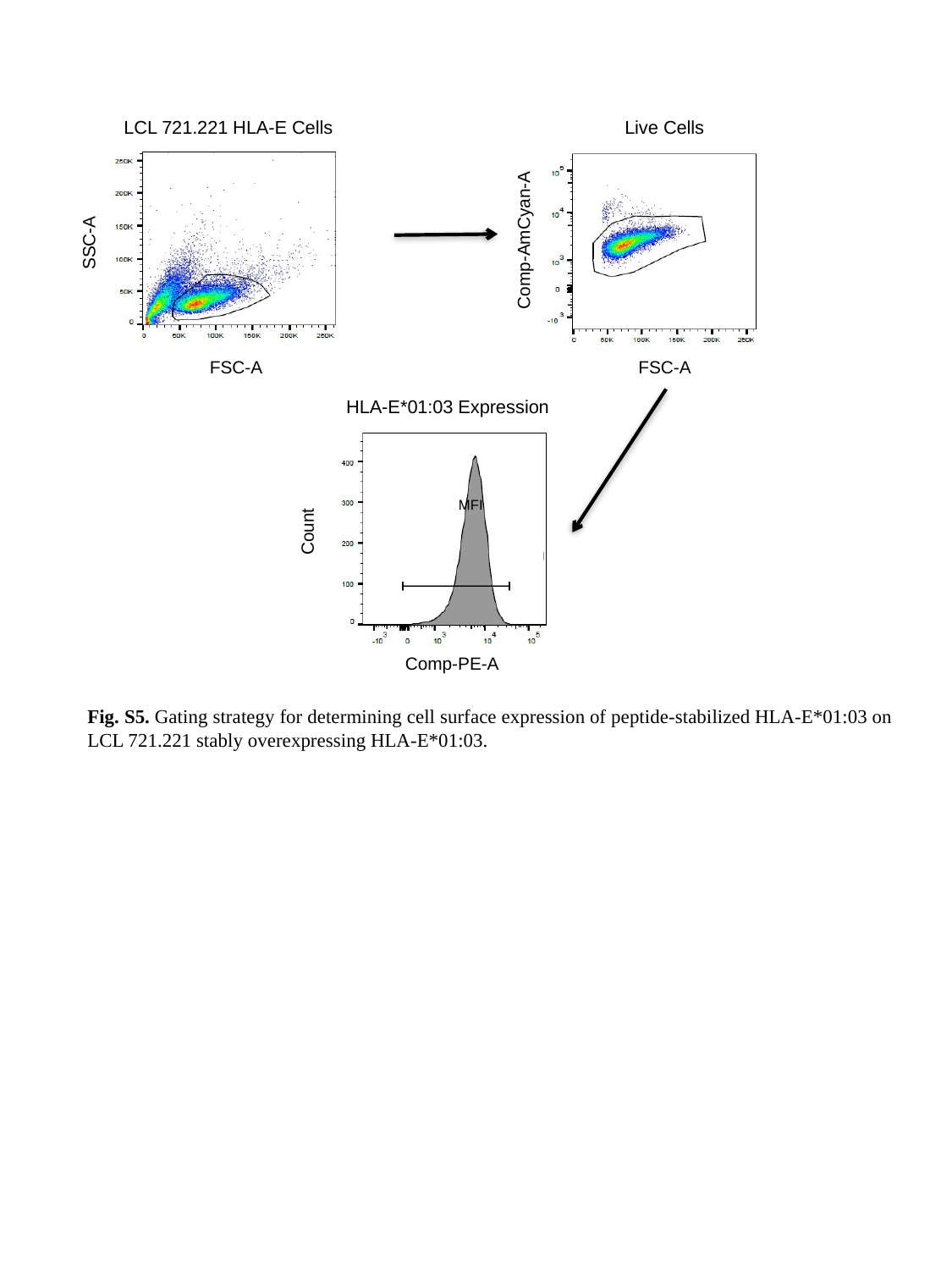

LCL 721.221 HLA-E Cells
Live Cells
Comp-AmCyan-A
SSC-A
FSC-A
FSC-A
HLA-E*01:03 Expression
MFI
Count
Comp-PE-A
Fig. S5. Gating strategy for determining cell surface expression of peptide-stabilized HLA-E*01:03 on LCL 721.221 stably overexpressing HLA-E*01:03.

## Slide 6
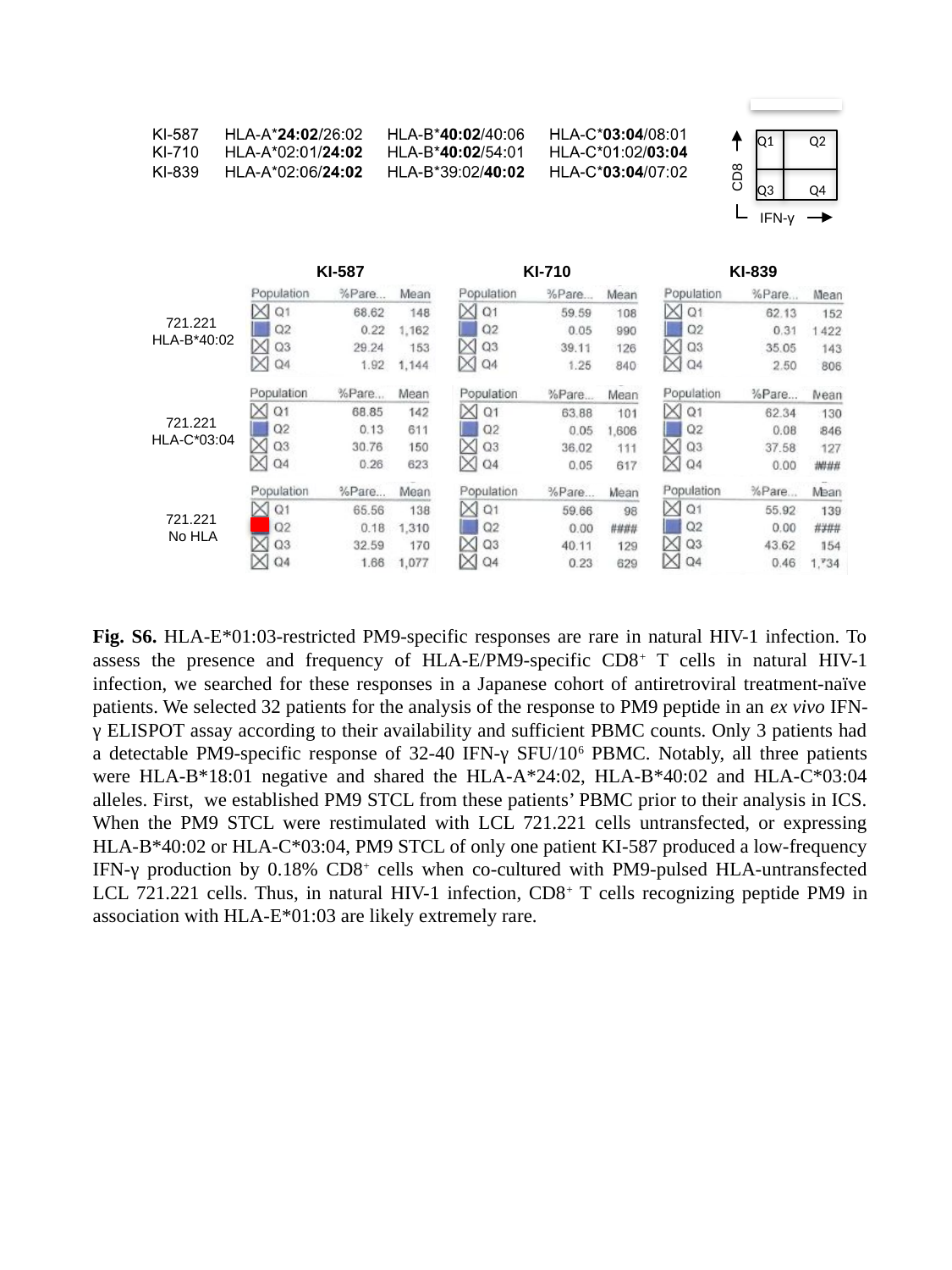

Q1
Q2
CD8
IFN-γ
Q3
Q4
KI-587
KI-710
KI-839
721.221
HLA-B*40:02
721.221
HLA-C*03:04
721.221
No HLA
Fig. S6. HLA-E*01:03-restricted PM9-specific responses are rare in natural HIV-1 infection. To assess the presence and frequency of HLA-E/PM9-specific CD8+ T cells in natural HIV-1 infection, we searched for these responses in a Japanese cohort of antiretroviral treatment-naïve patients. We selected 32 patients for the analysis of the response to PM9 peptide in an ex vivo IFN-γ ELISPOT assay according to their availability and sufficient PBMC counts. Only 3 patients had a detectable PM9-specific response of 32-40 IFN-γ SFU/106 PBMC. Notably, all three patients were HLA-B*18:01 negative and shared the HLA-A*24:02, HLA-B*40:02 and HLA-C*03:04 alleles. First, we established PM9 STCL from these patients’ PBMC prior to their analysis in ICS. When the PM9 STCL were restimulated with LCL 721.221 cells untransfected, or expressing HLA-B*40:02 or HLA-C*03:04, PM9 STCL of only one patient KI-587 produced a low-frequency IFN-γ production by 0.18% CD8+ cells when co-cultured with PM9-pulsed HLA-untransfected LCL 721.221 cells. Thus, in natural HIV-1 infection, CD8+ T cells recognizing peptide PM9 in association with HLA-E*01:03 are likely extremely rare.
